# Supplementary material for: Global Transcriptional Response of Methylorubrum extorquens to Formaldehyde Stress Expands the Role of EfgA and Is Distinct from Antibiotic Translational Inhibition
Source: Microorganisms. 2021 Feb 10;9(2):347. doi: 10.3390/microorganisms9020347 (PMC7916467; doi:10.3390/microorganisms9020347)
Supplement: Supplementary file 1 [file microorganisms-09-00347-s001.zip › microorganisms-1088209-supplementary-for proofreading/Bazurto et al F response-Microorganisms Supplemental figures-REVISION.docx]

**
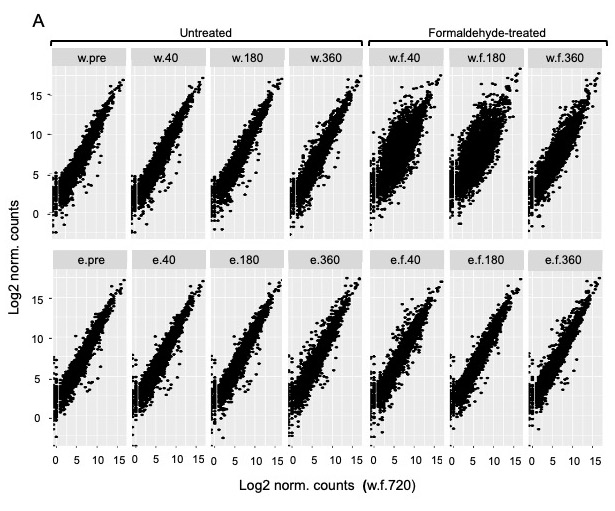
**

**Figure S1. Single replicate screen of transcriptional response identifies samples of interest.** A) Single replicate data for RNA-Seq of WT (‘w’, top panel) and Δ*efgA* mutant (‘e’, bottom panel) of *M. extorquens* grown in succinate minimal medium and untreated or treated with 5mM formaldehyde (‘f’) for 0 (‘pre’), 40, 180, 360, 720 min. Log2 transformed counts of the entire genome for each sample were plotted against those of WT formaldehyde-treated 720 min post treatment (w.f.720, x-axis) to establish the timescale of the transcriptomic response and identify the conditions most similar to the 720 min timepoint. B) Single replicate data for RNASeq of WT (‘w’, top panel) and Δ*efgA* mutant (‘e’, bottom panel) of *M. extorquens* grown in succinate minimal medium and treated with 50 μg/mL kanamycin (‘k’) for 40, 180, 360 min. Log2 transformed counts of the entire genome for each sample were plotted against those of WT pretreatment (w.pre, x-axis). Each point represents one gene.

**
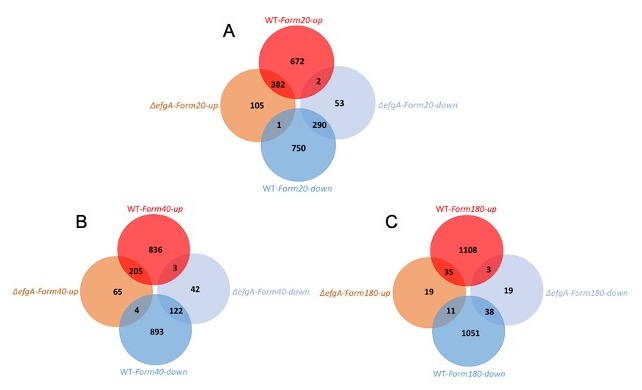
**

**Figure S2. Venn diagram of genes differentially expressed in WT and Δ*efgA* mutant upon formaldehyde treatment.** Each diagram represents a different timepoint A) 20 min, B) 40 min, C) 180 min. Genes differentially expressed (|log2-fold change| >1) were identified by dividing Log2FC of sample/Log2FC of pretreatment sample (of the respective genotype). Venn diagram for the earliest (5 min) timepoint is depicted in Figure 5.

**
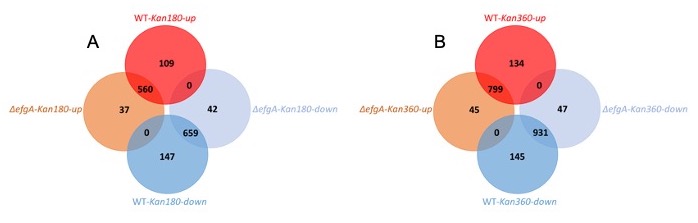
**

**Figure S3. Response to kanamycin is similar in WT and Δ*efgA* mutant.** Venn diagrams of genes differentially expressed in WT and Δ*efgA* mutant upon kanamycin treatment. Each diagram represents both genotypes at a distinct timepoint: A) 180 min and B) 360 min. Genes differentially expressed (|log2-fold change| >1) were identified by dividing Log2FC of sample/Log2FC of pretreatment sample (of the respective genotype).

**
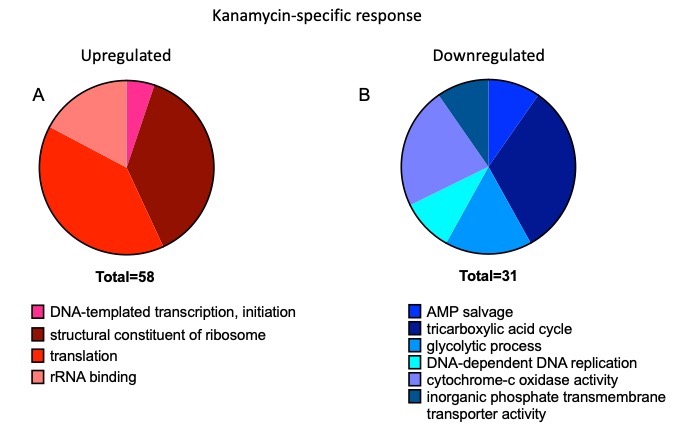
**

**Figure S4. Gene enrichment of the kanamycin-specific response.** Summary of functional group response of WT to kanamycin (360 min) was obtained using Comparative GO. All functional groups shown had >2 genes and p<0.05.

**
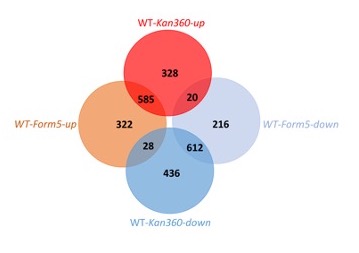
**

**Figure S5. Response to kanamycin and formaldehyde has overlapping genes.** Venn diagram of genes differentially expressed in WT upon formaldehyde treatment (5 min) or kanamycin treatment (360 min). Genes differentially expressed (|log2-fold change| >1) were identified by dividing Log2FC of sample/Log2FC of pretreatment sample (of the respective genotype). The 40 min kanamycin timepoint was excluded as expression profiles had not yet changed significantly.

**
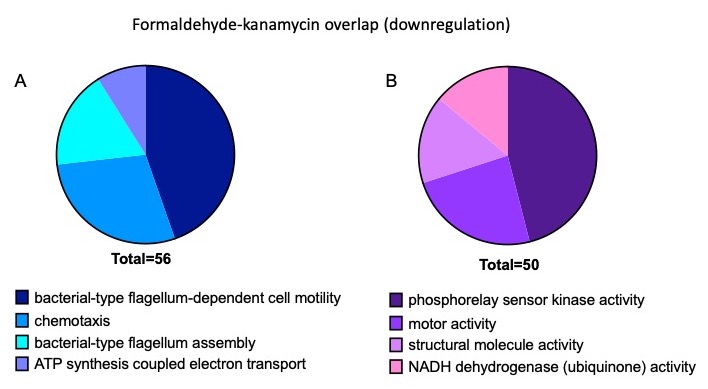
**

**Figure S6. Enrichment of downregulated genes in both the kanamycin and formaldehyde responses.** The overlap of gene functional groups in the downregulated responses of WT to kanamycin (360 min) and formaldehyde (5 min) was obtained using Comparative GO. Gene function is categorized by A) Biological Process B) Molecular Function. All functional groups shown had >2 genes and p<0.05.

**
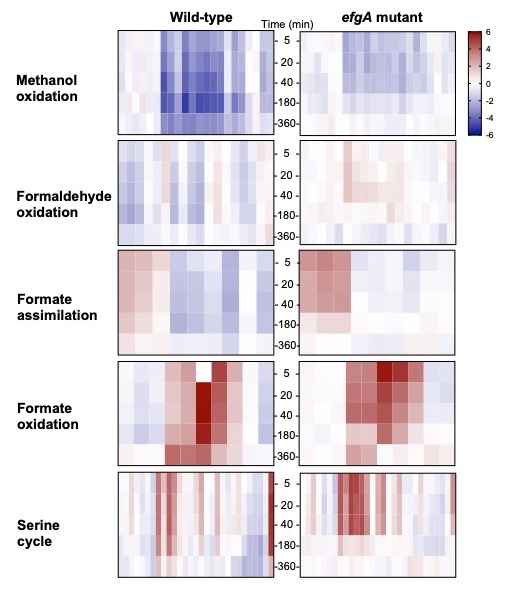
Figure S7. Temporal expression of genes of methanol utilization pathways.** Temporal heatmap of the Log2FC of all genes involved in methanol utilization pathways in WT (left) and Δ*efgA* mutant (right). Each horizontal pair of heatmaps represents genes involved in 1) methanol oxidation, 2) formaldehyde oxidation, 3) formate assimilation, 4) formate oxidation, and 5) serine cycle. Within each individual heat maps, each column represents a different gene and each row represents a particular time; organization is the same for both genotypes. Genes includes those involved in cofactor synthesis (PQQ, dH_4_MPT, T_4_H) and all formate dehydrogenases (FDH1, FDH2, FDH3, FDH4); individual gene locus tags and expression values are provided in the supplemental data table. The up or down directionality of expression change is indicated by the red or blue color gradient, respectively.

**
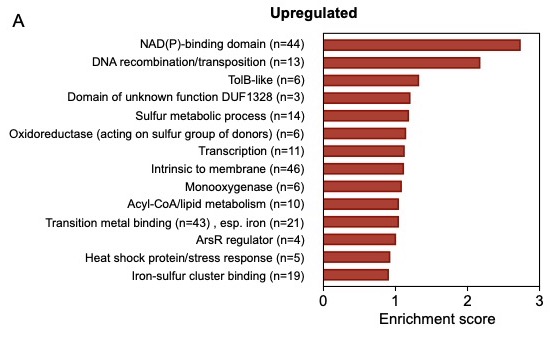

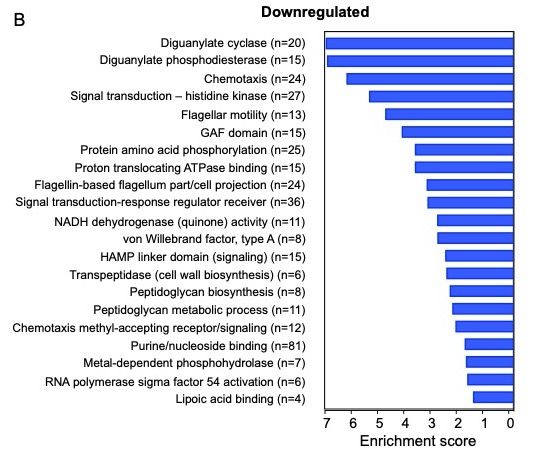
**

**Figure S8. Gene ontology of the early formaldehyde response of WT.** Summary of functional group response of WT at 5min (fastest response) and 20min (most intense response) was obtained using DAVID 6.7. Enrichment scores for each functional group is plotted for A) upregulated and B) downregulated genes; the number of genes in each functional group is indicated by ‘n’. All functional groups shown had p<0.05.

**
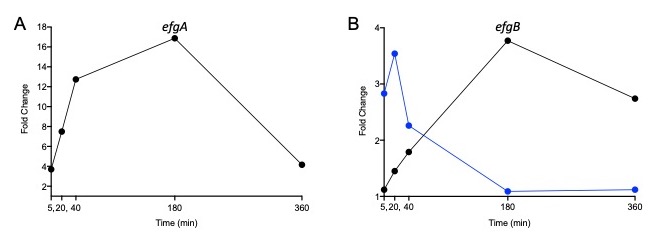
**

**Figure S9. *efgA* and *efgB* are upregulated in response to formaldehyde.** Temporal expression of A) *efgA* in WT and B) *efgB* in WT (black) and the *efgA* mutant (blue) in response to formaldehyde treatment. Log2FC of each gene is plotted.

**
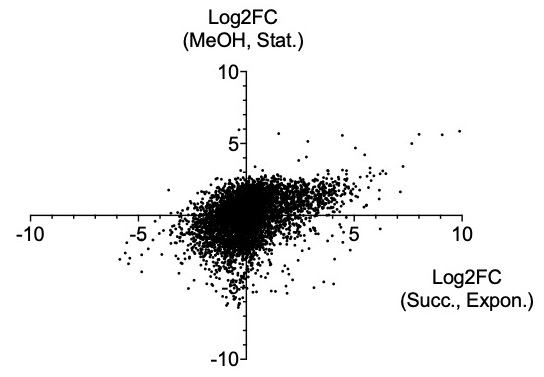
**

**Figure S10. Comparison of early formaldehyde response in different growth conditions.** Expression changes (Log2FC) of all genes at the earliest timepoint (5 min) here, where succinate-grown cells were treated with 5 mM formaldehyde in early exponential phase of growth are plotted against expression changes of the same genes from a previous experiment [[50]](https://paperpile.com/c/Siitmp/HQIvp) where methanol-grown cells were treated with 4 mM formaldehyde in stationary phase of growth and assessed at 4 hr.

**
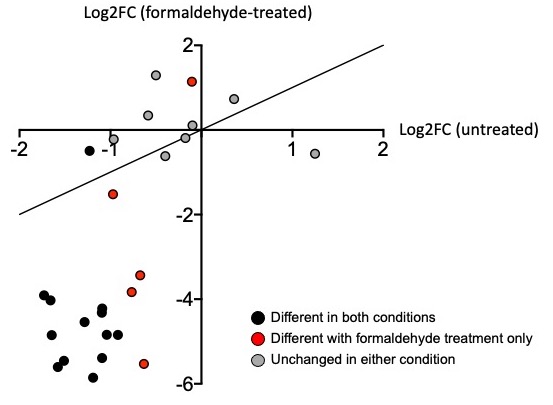
**

**Figure S11. Response of genes involved in formaldehyde tolerance heterogeneity.** Expression changes (Log2FC) of genes predicted to be differentially expressed in formaldehyde tolerant subpopulations were examined in untreated (x-axis) and formaldehyde-treated (y-axis) samples.

**
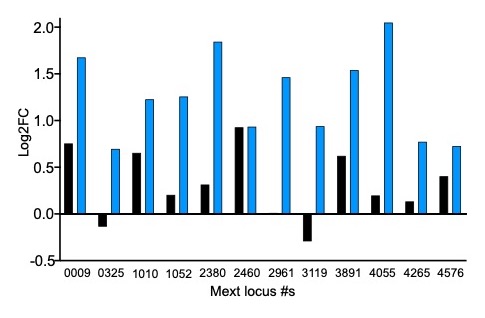
**

**Figure S12. Genes involved in Nuclease activity and DNA metabolism & repair suggest the Δ*efgA* mutant experiences different DNA stress than WT.** Expression of genes involved in nuclease activity and DNA metabolism & repair are shown for WT (black) and *efgA* (blue)*.* Log2FC of each gene in response to formaldehyde (5 min) is plotted.
